# WSES SM (World Society of Emergency Surgery Summer Meeting) highlights: emergency surgery around the world (Brazil, Finland, USA)

**DOI:** 10.1186/1749-7922-4-11

**Published:** 2009-03-30

**Authors:** Renato Poggetti, Ari Leppanemi, Paula Ferrada, Juan Carlos Puyana, Andrew B Peitzman, Luca Ansaloni, Fausto Catena, Antonio D Pinna, Ernest E Moore

**Affiliations:** 1 Emergency Surgical Service, Emergency Surgical Service Hospital das Clinicas, School of Medicine, San Paulo, Brazil; 2 Department of Surgery Meilahti hospital University of Helsinki, Helsinki, Finland; 3 University of Pittsburgh Medical Canter, Pittsburgh, USA; 4 General, Emergency and Transplant Surgery DPT St Orsola-Malpighi University Hospital, Bologna, Italy; 5 Department of Surgery, Denver Health Medical Center, University of Colorado Health Sciences Center, Denver, USA

## Abstract

Emergency surgery is performed in every hospital with a A and E unit all around the world. However it is organized in different ways with different results.

Aim of this paper is to present history, current scope, current training program and new politics for training national program of 3 countries of different continents.

Brazil, Finland and US emergency surgery models are presented discussing all criticisms showed during the WSES Summer Meeting 2008.

## Emergency Surgery in Brazil

### Modern History

Trauma is the second cause of death in Brazil killing more than 130.000 people per year. Emergency surgery is also a health problem because many surgical diseases are not diagnosed earlier allowing the onset of complications that require emergency surgical treatment. On the other hand the health ministry has defined trauma and all emergencies as priority areas of interest in Brazil and has invested in improvements as the pre hospital care system in the whole country. Traditionally trauma and emergency surgery were always treated together in the emergency department of public general hospitals in Brazil. Until now great progresses have been obtained by the Brazilian surgical community with the intense experience of the emergency departments and the development of new surgical techniques, thanks to the ability of improvisation and the great creativity of the Brazilian surgeons. Programs like ATLS are spread in the entire country. Others like the PHTLS are growing actively. During the last two decades the pre hospital care system that didn't exist, grew quickly and now covers around 800 cities and 50% of the country population. On the other hand, as for organization and the system development levels we are still sprouting. The Brazilian Trauma Society, a medical society that congregates surgeons and other professionals of trauma care only now is getting independent and self maintained. The Committee on Trauma of the Brazilian College of Surgeons is also starting to march towards the establishment of local protocols and patterns for the surgeon that works in the emergency department. There is a lot to do. We have no national data bank and there is no specific residency program for the trauma and emergency surgeon. We don't have a program for certification and verification of trauma and emergency surgery centers in the country and national protocols for definitive treatment of trauma and emergency surgery are still lacking. [1]

### Current Scope of practice

Medical care in Brazil is mainly provided through a socialized medical system that offers free medical care to the entire population. A relatively small percentage of the population has access to private medical care. Emergency medical care is free of charge even for people that have private medical care insurance. The hospital reimbursement for emergency medical care of the socialized system is low and does not cover the real costs. The emergency rooms and services are overcrowded which compromises the quality of the care delivered. The private medical care system values more the specialists than the generalist medical professionals. This scenario reinforces the tendency of the medical students to become specialists rather than generalists. Our health system has no emergency physician specialists that are trained and work exclusively in emergency services. The emergency care is delivered by a group of specialists that work together in shifts of 12 or 24 hours in the emergency services throughout the country. This policy requires many physicians working simultaneously which increases the cost of the man power. This circumstance creates a unique situation where the surgeon specialist has at least two distinct activities: during the day he is a specialist and at night and weekends he works as trauma and as emergency surgery specialist.

Intensive care medicine is a specialty. The man power force is built up mainly by anesthetists and physicians that after training at least two years on their specialty, spend another two years more on the intensive care residency program. The total number of UCI beds is not enough for the departments of trauma and emergency surgery.

Pre hospital care has been growing and getting better organized during the last two decades. There is still a lot to work in this area because more than half of the population is not yet covered by the system. Every day the pre hospital care system is bringing in more severe trauma patients to hospital care. On the other hand the trauma surgeon needs to be better prepared to treat the more severe traumatized patient.

Traditionally the trauma surgeon works in house during shifts of 24 hours. He performs trauma and non-trauma emergencies. The surgeon does not work, neither covers the ICU because intensive care medicine is a specialty. Vascular surgery is performed by vascular surgeons trained with two years of general surgery and two years of vascular surgery.

How is Acute Care Surgery in Brazil now? Trauma and emergency surgery are performed by surgeons with two years of general surgery training, or by specialists with two years of general surgery training and two or three years of an another specialty training. The surgeon is usually hired to work 24 hours per week in a public hospital and he can be hired simultaneously by two different public hospitals. The majority of these surgeons work on shifts of 24 hours in one or two different hospitals. Trauma and emergency surgery are treated by surgeons that are working in 24 hours shifts and some hospitals, but not all, have surgeons that work every day in the same hospital in a horizontal fashion, taking care of the patients after the first surgery that was performed in the emergency department. The damage control technique is frequently used but the follow up of the patient and the subsequent surgical procedures are not necessarily done by the same surgeon that performed the first procedure. In this scenario the trauma and emergency surgery doctor is not motivated for trauma and emergency surgery care because of at least four pivotal reasons: 1) he is not well prepared, 2) he is not certified as a surgeon of trauma and emergency surgery, 3) this activity is not his main area of interest, and 4) this is not a well defined area of activity in the context of the Brazilian medical care system. [2]

### Current training program

Basic education in Brazil is built up of four years of elementary school, four years of intermediate school and three years of high school. Usually you need to spend one extra year of intense studying program to be approved in a formal selective exam to be admitted to a medical school. The better the medical school, the more difficult it is to get in. The best medical schools of the country are public and free of charge and consequently the students of wealthy families that can afford to be prepared in private schools during their basic education of eleven years, have better chance to get into a good medical school. The medical course lasts six years. Brazil has around 150 medical schools, with an average of 100 students per school, per year, for a population of 184 million people. The quality control of the schools is not very rigorous and some medical schools do not have their own hospitals for clinical rotations of the medical students. The distribution of these 150 medical schools is not uniform so you have some regions of the country with many schools and other areas with very few schools. Trauma and emergency surgery are not formally taught in the curriculum of all medical schools so, many doctors finish their graduate course without a good knowledge of emergency surgery and trauma care.

The following aspects must be considered when you analyze the surgical residency. In order to become a general surgeon in Brazil, the doctor has to do only two years of general surgery residency program. According to Brazilian laws, at the end of two years of general surgery residency, the medical doctor is certified as a general surgeon and can practice emergency surgery and trauma care in the entire country. In an attempt to improve general surgery training, some residency programs in the country divided the general surgery residency program in two sections: basic general surgery of two years and advanced general surgery of two more years. The other surgical specialties require two years of general surgery and two or three years of the specific surgical specialty residency program. After two years of general surgery residency the hospitals and the government certify the doctors as general surgery specialist. The governmental organizations think that it is sufficient to train a general surgeon during two years for him to work in the medium and small size towns in the country. This surgeon will work taking care of general surgery and trauma and emergency surgery. All specialist surgeons in Brazil have two titles, general surgeon and another specialty, for example, cardiac surgeon, vascular surgeon, etc. The majority of general surgery residency programs in Brazil have only two years of surgical training, and only few programs offer four years of general surgery training. There are not enough places in the residency programs for all medical students that come out of the medical schools each year. The quality control of the residency programs in the country still requires improvement. There is a culture of valorization of the specialist in detriment of the generalist doctor. Finally, the geographic distribution of the residency programs gives priority to the larger populated urban areas. After finishing the residency program the doctors prefer not to go to the rural or less populated regions of the country.

### New Politics for Training National Program – Future Directions

After analyzing the previous topics we easily conclude that there is a need in Brazil for the Acute Care Surgeon responsible for the care of trauma and emergency surgery. It is also clear that this area of activity needs to be well defined, developed, preserved and protected by a medical society. It is very important to understand how medical profession specialties and medical training programs are organized and related in our country. Brazil has 53 specialties that are connected to their respective societies. Residency programs for these specialties must have two years of training program and well defined previous requirements. As so, these specialties establish residency program and determine the number of trainees that will receive financial support to do the residency program. The support comes from the government. The organizations that regulate all these activities are the Brazilian Medical Society ("Associação Médica Brasileira" – AMB), the Federal Council of Medicine ("Conselho Federal de Medicina" – CFM) and the National Council of Residency Program ("Conselho Nacional do Programa de Residência" – CNPR). Together they compose a Joint Commission (Comissão Mista – CM) that approves new specialties and new residency programs. In order for an area of medical activity to become a specialty that area needs to be of social interest, recognized by the health ministry, and it needs to be supported by the medical society that shelters that area of medical activity. After an approval of the CM, the government gives financial support for the program and the residents that will attend the program. The government also recognizes the certification of the residency program.

Trauma and emergency surgery have a very high prevalence in Brazil and medical societies, the government and health ministry recognize it. The Brazilian Trauma Society (Sociedade Brasileira de Atendimento Integrado ao Traumatizado – SBAIT) is the medical society responsible for trauma and emergency surgery in Brazil. SBAIT is still sheltered by the Brazilian College of Surgeons (Colégio Brasileiro de Cirurgiões – CBC). The Brazilian College of Surgeons has already recognized that trauma and emergency surgery need to become a specialty under the patronage of SBAIT. Now SBAIT needs to seek approval of AMB, CFM, CNRM, CM and establish the trauma and emergency surgery (Acute Care Surgery) residency program around the country. SBAIT knows that there are more than ten centers in the country that can and want to implement trauma and emergency surgery residency programs now. After implementation of the residency program, the SBAIT and the governmental organizations will be responsible for certification and quality control of the programs and the centers that offer the programs. I certainly believe that this is going to be a pivotal step in the development and improvement of Acute Care Surgery in Brazil. Once this step has been attained subsequent challenges certainly will be more naturally defeated. [2]

## Emergency surgery in Finland

### Modern history

After being part of the kingdom of Sweden-Finland for more than 400 years and subsequently an autonomous area belonging to the Russian empire, Finland gained independence in 1917. It participated in the Second World War by preventing a Soviet invasion. The social structure is very similar to other Scandinavian countries and is characterized by a well-developed and tax-funded health care and social welfare system.

The population of Finland is 5.2 million living in an area of 337.000 km2 (roughly the size of Italy or Great Britain). In the beginning of 2003, there were 19.764 registered physicians in Finland (263 inhabitants/physician), of which 42% worked in public hospitals and 20% in primary healthcare centers. Sixty percent of the physicians were specialists and 20% had a Ph.D.-degree.

The per capita GDP is USD 26.200, the infant mortality rate 3.8/1000 live births, and life expectancy 77.8 years (81.5 for women and 74.1 for men). Of the total of 48.504 deaths in 2001, 4166 (9%) were caused by accidents and violence (80 deaths/100.000 inhabitants/year). Of the 2651 accidental deaths (64% of all deaths caused by accidents and violence), 39% were caused by falls, 22% by accidental poisonings, 20% by traffic accidents and 5% by drowning. There were 1204 suicides (29% of all deaths caused by accidents and violence), and 154 deaths (4%) caused by violence. Knives cause the majority of penetrating injuries. The annual incidence of firearm injuries in Finland is about 6.2/100.000 inhabitants.

### Current scope of practice

There are 5 medical schools built around university hospitals in Finland. In the multi-layered public health care system, primary care is provided by the healthcare centers in each of the about 400 counties. For specialist care, Finland is divided into 21 hospital districts. There are 5 university hospitals and 16 central hospitals that provide most of the specialist care including surgical emergency services in their respective areas. There are also some, smaller district hospitals where basic surgical services are provided. The 5 university hospitals have special responsibilities for the most demanding specialized care in their area, and in some cases, such as transplantation surgery or major burn care, the centralization goes even further to one or two centers in the whole country. Overall, about 400.000 surgical procedures are performed each year in the public hospitals. In addition, there are private hospitals mostly in larger cities providing elective surgical services with varying degree of specialization.

The majority of patients with emergency surgical problems are managed in the university and central hospitals, although certain specialist services, such as cardiothoracic and neurosurgery, are available almost exclusively in university hospitals. In most central hospitals, there are usually one or two surgical residents on call in the hospital outside the working hours with more senior surgeons (one general or "visceral", and one orthopedic surgeon) on call at home with a response time obligation of 30 minutes at the most. The university hospitals have usually in-house specialist surgeons from the large specialties (gastroenterological surgery, orthopedics and traumatology) available around the clock and on-call services from home of other specialties including urology, vascular surgery, pediatric surgery, plastic surgery etc.

Most of the emergency general surgery (mainly acute abdomen) is performed by gastroenterological surgeons or residents, but in smaller hospitals also other visceral surgeons (urologists or vascular surgeons, for example) participate in the on-call rosters. Same applies to abdominal trauma including damage control surgery, whereas vascular or thoracic trauma patients are usually referred to a center with vascular surgeons or thoracic surgeons on-call, respectively. Intensive care is largely provided by anesthesiologists with special interest in intensive care. Intensive care is not part of the surgical training curriculum.

### Current training program

In the past, all surgeons were trained as general surgeons (including orthopedic surgery) for 6 years. If desired, an additional two-year fellowship in some specialized field (gastroenterological surgery, urology, plastic surgery, orthopedic surgery etc.) could be taken leading to a subspecialty in that field. In the law on medical education form 1999, many of the previous subspecialties were changed into main specialties reducing the overall number of specialties from 92 to 49, and shortening the duration of all training programs to 5 or 6 years. In surgery, a common trunk program of 3 years, which includes a 9-month primary health care rotation, was designed to familiarize the resident with basic surgical techniques while working in a central or district hospital under the supervision of a more senior surgeon and learning to perform independently the more common basic surgical emergency operations such as appendectomies, incarcerated hernia operations, fixation of ankle fractures etc.

After the common trunk period, another 3-year period in one of the university hospitals is required in one of the following fields: gastroenterological surgery, cardiothoracic surgery, vascular surgery, urology, orthopedics and traumatology, hand surgery, plastic surgery, pediatric surgery, and general surgery. The new law created 2 new specialties, vascular surgery (separated from cardiothoracic surgery) and general surgery (an independent specialty). Oral and maxillofacial surgery and neurosurgery are also main specialties with a 6-year training program but are not following the common trunk training program of other fields of surgery. Theoretical education of 100 hours and a national examination are part of all specialization programs.

There is no emergency surgery specialty in Finland. Surgeons specialized in orthopedics and traumatology look after most of the polytrauma patients, whereas visceral injuries are largely managed by organ-specific specialists, at least in bigger hospitals.

### Future directions

The current specialization system is in harmony with the European Union requirements and will guarantee the supply of well-trained surgeons for specialized elective surgery. However, it is seriously deficient in providing surgical competence for managing acute surgical problems, in terms of knowledge, decision making and technical skills. General surgical knowledge and skills are eroding rapidly and this has caused great concern among the surgical profession in Finland. Inevitably this will lead to increasing centralization of trauma and emergency surgery services, a trend that is already visible in many parts of the country.

A new law on medical education is under preparation and will probably be effective within the next 1–2 years. Among other things, it lengthens the common trunk period with one year, and effectively the overall training period from 6 to 7 years. It also seems to end the role of general surgery as an independent specialty. Whether this will alleviate the problems associated with the current training system is questionable.

The Finnish Society of Surgery has taken the initiative to urge for complete reorganization of the surgical services based on a regionalized model. The emergency surgery services would be provided by five regions built around the university hospitals that would act as the single (level-1) trauma and emergency surgery center for the whole region. Because of long distances especially in the northern and eastern parts of the country and the larger population bases in the southern and western parts, most regions would have another (level-2) emergency surgery center that would provide most of the surgical specialist services for the nearby population with the exception of cardiothoracic and neurosurgery. Major burns would be centralized into one burn center in the whole country.

Finally, who would lead the multidisciplinary team managing polytrauma and other complex surgical patients that might require intervention of multiple specialists including interventional radiologists and endoscopists? An appropriately trained surgeon with expertise in trauma and emergency surgery, good decision making skills and the technical ability to perform a large part of the life- and limb-saving surgery required during the first 24 hours could act as the hospitalist surgeon and first-line defense, and be a mentor and team leader synchronizing the work of other specialists. In addition, a surgeon trained in emergency surgery would be an ideal person to run and develop trauma and emergency surgical units in larger hospitals as well as plan for mass casualty situations.

## Emergency Surgery in the United States

### Modern History

In the United States, approximately 1000 general surgeons complete their residency training each year. Seventy percent of graduating surgical residents currently pursue fellowship surgery training, most commonly in colorectal or laparoscopic surgery. [3]

This increased trend toward subspecialization confounds work force projections. Available databases provide only an estimate of the extent of this trend. When surgeons complete fellowships, they narrow the spectrum of services provided.

There are many reasons why surgical residents decide to specialize. One of them is monetary reimbursement. By the time of graduation, general surgery residents have completed 4 years of college, 4 years of medical school, and close to 5 years of residency depending on the area of specialization chosen. Trainees with academic aspirations spend multiple additional years in a research laboratory during their residency years.[4] Life styles and large debts on educational loans may also influence the decision for the pursuit of further training. In addition, with continued specialization of surgery, many graduates feel that fellowship training is required for them to become competent in their area of interest.

The now classic report by Miller and Richardson, soliciting the opinions of senior residents about their perspective of trauma surgery was telling. Eighteen percent of the senior residents thought they may do some trauma surgery in their practices. Few had positive views of trauma surgery as a career – undesirable clientele, lifestyle, too much nonoperative work, lack of elective general surgery, and they did not view the trauma surgeons as part of general surgery. This lack of positive role-modeling is a critical issue. The residents did not think trauma surgeons were "real" general surgeons.

Trauma care has evolved in the last 20 years. During the 1980's, there was an increase of penetrating injuries in the United States. Also, the management of blunt abdominal injury was largely operative. With the evolution of technology and radiological adjuncts, many of the injuries that were managed with surgery had a better outcome while being managed conservatively. This change decreased the amount of procedures that a surgeon dedicated to trauma could perform.

Acute care surgery is not a new concept. In many areas of the USA, the general surgeon cares for all trauma patients and patients with surgical emergencies, especially in rural areas. In many instances, these individuals are the workforce of the hospital, and the most important source of income for the institution.

### Current Scope

The concept of Acute Care Surgery was born many years before it was recognized as a specialty because of need. The need to have further specialized training in general surgery, the need to have an appropriated reimbursement to individuals dedicated to this discipline, the need to train surgeons to take care of emergencies with proficiency, and to recognize the immense and growing demand for emergency and critical care surgical coverage.

The population of general surgeons is decreasing. Fewer residents are choosing general surgery and existing general surgeons are aging. As a result, 32% of general surgeons are older than 55 years and 20% are younger than 35 years of age.[5] Emergency department visits have increased 26% since 1993, and 75% of hospitals report inadequate on-call surgeon coverage.

In several institutions, the trauma surgeon for years has been the individual who provides care for the patients coming to the emergency room. In rural hospital, the general surgeon fills this role. This includes all types of emergencies: vascular, emergent laparotomies, cholecystectomies, appendectomies and treatment of abdominal catastrophes such as bleeding obstruction or perforations. It is mostly in large academic centers where the thoracic and vascular cases are treated by specialist in each field.

### Current Training Program

The American Association for the Surgery of Trauma (AAST) in conjunction with the American College of Surgeons, took the initiative to develop this fellowship considering the problems of patient access to emergency surgical care and the future viability of trauma surgery as a career.[6]

The three major components of Acute Care Surgery are: Surgical Critical Care, Trauma and Emergency Surgery.

The curriculum includes at least six months of critical care and 15 months of elective and emergency surgery.

The surgical rotations include trauma, thoracic, hepatobiliary, vascular, orthopedic and neurological surgery.

The intention of this design is to train a surgeon to provide care for patients based on disease processes. A trauma/acute care surgeon should be able to care for any mechanical injury independently of the organ system were the injury is present.

The infrastructure of large academic programs precludes the general surgeons from providing operative care of orthopedics or neurosurgical issues. The intention in these cases is a better understanding of the decision making and disease process behind the injury and treatment.

### New policies of Training

While completing the acute surgery fellowship, the trainees must participate in acute care surgery call no less than 12 months. Flexibility is essential in the timing of these rotations, and the structure of the 24-month training, should be utilized to optimize the fellow's preparation.[7]

The Acute Care Surgery fellowship sites must have an RRC-approved SCC residency, where the participation in elective surgery will be an essential component of the fellowship training. Most importantly, an academic environment is mandatory and fellows should be trained to teach others and conduct research in acute care surgery.

For Acute Care Surgery to be attractive and a sustainable field, structural changes must occur: 1. Job satisfaction: The complexity and number of cases will need to be satisfactory, as well as the appropriate reimbursement. 2. The specialty must be recognized and respected by our surgical peers. For this field to be attractive to residents, the lifestyle must be an important aspect of how we redesign the specialty. A critical mass of partners is necessary to ensure that there is time for other activities such as research education, administration as well as leisure and recreational; activities or good quality time with families exist in order to maintain the practice.

## Competing interests

The authors declare that they have no competing interests.

## Authors' contributions

RP wrote Emergency Surgery in Brazil. AL wrote Emergency Surgery in Finland. PF wrote Emergency Surgery in US. JCP wrote Emergency Surgery in US. ABP wrote Emergency Surgery in US. LA conceived of the study, and participated in its design and coordination. FC conceived of the study, and participated in its design and coordination. ADP conceived of the study, and participated in its design and coordination. EEM conceived of the study, and participated in its design and coordination. All authors read and approved the final manuscript.
